# The Dynamic Relationship of Breast Cancer Cells and Fibroblasts in Fibronectin Accumulation at Primary and Metastatic Tumor Sites

**DOI:** 10.3390/cancers12051270

**Published:** 2020-05-17

**Authors:** Sarah Libring, Aparna Shinde, Monica K. Chanda, Maryam Nuru, Heather George, Aya M. Saleh, Ammara Abdullah, Tamara L. Kinzer-Ursem, Sarah Calve, Michael K. Wendt, Luis Solorio

**Affiliations:** 1 Weldon School of Biomedical Engineering, Purdue University, West Lafayette, IN 47907, USA; slibring@purdue.edu (S.L.); mchanda@purdue.edu (M.K.C.); mnuru@purdue.edu (M.N.); georgeh@purdue.edu (H.G.); saleh0@purdue.edu (A.M.S.); tursem@purdue.edu (T.L.K.-U.); sarah.calve@colorado.edu (S.C.); 2 Department of Medicinal Chemistry and Molecular Pharmacology, Purdue University, West Lafayette, IN 47907, USA; shinde.aparna014@gmail.com (A.S.); abdulla6@purdue.edu (A.A.); 3 Purdue Center for Cancer Research, Purdue University, West Lafayette, IN 47907, USA

**Keywords:** fibronectin, fibroblast, epithelial mesenchymal transition, plasticity, breast cancer, extracellular vesicle, premetastatic niche, metastasis

## Abstract

In breast cancer (BC), tissue stiffening via fibronectin (FN) and collagen accumulation is associated with advanced disease progression at both the primary tumor and metastatic sites. Here, we evaluate FN production in 15 BC cell lines, representing a variety of subtypes, phenotypes, metastatic potentials, and chemotherapeutic sensitivities. We demonstrate that intracellular and soluble FN is initially lost during tumorigenic transformation but is rescued in all lines with epithelial-mesenchymal plasticity (EMP). Importantly, we establish that no BC cell line was able to independently organize a robust FN matrix. Non-transformed mammary epithelial cells were also unable to deposit FN matrices unless transglutaminase 2, a FN crosslinking enzyme, was overexpressed. Instead, BC cells manipulated the FN matrix production of fibroblasts in a phenotypic-dependent manner. In addition, varied accumulation levels were seen depending if the fibroblasts were conditioned to model paracrine signaling or endocrine signaling of the metastatic niche. In the former, fibroblasts conditioned by BC cultures with high EMP resulted in the largest FN matrix accumulation. In contrast, mesenchymal BC cells produced extracellular vesicles (EV) that resulted in the highest levels of matrix formation by conditioned fibroblasts. Overall, we demonstrate a dynamic relationship between tumor and stromal cells within the tumor microenvironment, in which the levels and fibrillarization of FN in the extracellular matrix are modulated during the particular stages of disease progression.

## 1. Introduction

Breast cancer (BC) is the most frequently diagnosed cancer among females and the second leading cause of cancer deaths [1]. Metastasis accounts for the vast majority of patient mortality [2,3]. During disease progression, distinct changes occur in the extracellular matrix (ECM) architecture and biochemical composition, which facilitate primary tumor growth and successful metastasis [3,4]. The glycoprotein fibronectin (FN) is of particular interest as FN expression in primary mammary tumors is strongly correlated with decreased patient survival for all BC subtypes [5,6].

In the primary tumor, FN and collagen fibrils accumulate as the tumor develops, which causes an increase in tissue stiffness. This matrix stiffness promotes proliferation, increases tumor cell aggressiveness, and is believed to bolster the number of cancer stem cells [4,7]. Recent research has indicated that FN and collagen fibrils accumulate parallel to the primary tumor border during premetastatic growth. However, in invasive tumors, these fibrils realign perpendicular to the tumor border, acting as “tracks” for tumor cell migration through the basement membrane [7,8,9].

Growing evidence suggests that the primary tumor also primes distant organs in the development of a premetastatic niche to aid metastasis [10,11]. In mouse models, FN deposition is upregulated in premetastatic niches [4,9]. This increased FN content is believed to aid disseminated cancer cell recruitment to the secondary organ and may also promote metastatic initiation after extravasation [4,12,13,14]. Extracellular vesicles (EVs), including exosomes, have recently been shown as a key source of endocrine communication from the primary tumor [15,16,17,18]. In particular, EVs have been reported as a major cause of FN accumulation in the premetastatic niche. They have also been connected to the recruitment of tumor-supporting stromal cells and the enhanced attachment of disseminated tumor cells [10,19]. We recently demonstrated that conditioning pulmonary fibroblasts with EVs from BC cells could drastically alter the subsequent growth of epithelial BC cells in a FN-dependent manner [14]. 

Currently, it is unclear what role BC cells have in the accumulation of extracellular FN. Soluble FN is found extensively in the plasma (300 µg/mL) [5,6]. However, FN in solution has a compact confirmation and will not self-assemble into fibrils as a function of concentration [20,21,22]. Instead, fibrillarization of FN to form a matrix requires an exogenous stimulus. The FN must be stretched to expose the cryptic FN–FN binding site and enable fibrillar matrix assembly. This typically occurs through cell-mediated processes via integrin binding (primarily the α5β1 integrin) to the Arg-Gly-Asp (RGD) domain [20,21,22,23]. Because FN assembly is a multi-stage process, BC cells may secrete increased levels of soluble FN, may be capable of organizing FN fibrils, or may contribute to increased FN matrix production by the non-cancerous cells in the tumor microenvironment, [24]. Recent studies by our group suggest that breast cancer cells that undergo epithelial–mesenchymal transition (EMT) can produce large amounts of globular FN, but they do not actively participate in matrix deposition [6]. 

In this work, we compared the accumulation of FN at the primary tumor and in lung metastasis using histology and tissue clearing techniques. We systematically compared intracellular and extracellular FN levels among BC cell types with varied phenotypes, metastatic potentials, and drug sensitivities using immunoblot analysis and decellularization techniques [4,6]. We found phenotypic-dependent FN deposition by conditioned fibroblasts, with differential responses when modeling paracrine and endocrine signaling. Overall, our findings shed light on site-dependent and phenotype-dependent fibroblast conditioning, resulting in differential FN matrix accumulation as it relates to changes in the tumor microenvironment throughout primary tumor development, invasion, and macrometastasis. 

## 2. Results

### 2.1. Fibronectin Accumulates Transiently In Vivo

**Figure 1:**
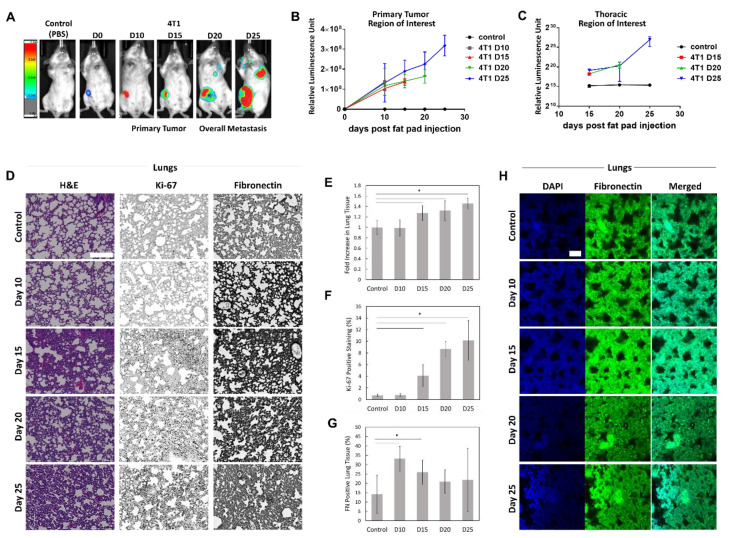
(**A**) Primary tumor growth and overt metastasis of 4T1 breast cancer cells, tracked using luminescence, following mammary fat pad injection. Luminescence values (**B**) steadily increased in the primary tumor for each time point and (**C**) showed overt pulmonary metastasis occurring between days 20 and 25. Control mice were sacrificed at day 25. (n = 3 mice, mean ± s.d.). (**D**) Representative H&E, Ki-67, and fibronectin (FN) staining of 5 µm lung sections at each time point. Scale bar is 200 µm. (**E**) The lungs showed a loss of native structure with an accumulation of tissue density, quantified from H&E staining (*n* = 6 images, mean ± s.d.). (**F**) Ki-67 positive cells significantly increased in tumor bearing mice starting at day 15 (*n* = 6 images, mean ± s.d.). (**G**) FN levels initially increased but returned to control levels by day 20 (*n* = 6 images, mean ± s.d.). (* indicates *p* < 0.05). (**H**) Cleared whole lobes confirm tissue accumulation. Scale bar is 50 µm.

To establish FN accumulation during disease progression, we injected 4T1 BC cells expressing luciferase into the mammary fat pad of BALB/c mice and tracked primary tumor growth and lung metastasis via luminescence over 25 days (Figure 1A). Luminescence increased as expected in the primary tumor over time (Figure 1B). Small differences were quantified in thoracic luminescence levels from day 15, but overt metastases were not seen in any mice until day 20, and not consistently until day 25 (Figure 1C). Whole tumors were cleared, and immunostaining revealed an increase in FN expression and overall density over time. The edges of the tumor also appeared serrated on days 20 and 25, indicating possible infiltration into the surrounding healthy tissue (Figure S1). H&E staining of lung sections indicated an accumulation of extracellular tissue, disrupting the natural honeycomb structure seen in control lungs (Figure 1D). There was a significant increase in lung tissue density from day 15 onward as compared to nontumor bearing mice that were sacrificed on day 25 (Figure 1E). Similarly, there were significantly more Ki-67 positive cells in the lung tissue starting on day 15 (Figure 1D,F). FN staining of the lung tissue indicated a transient increase, which was significant on days 10 and 15, before falling to a level that was not significantly different than control mice (Figure 1D,G). Cleared whole lobes were used to confirm changes in FN accumulation and tissue volume (Figure 1H).

### 2.2. Fibronectin Is Not Fibrillarized by Breast Cancer Cells

**Figure 2:**
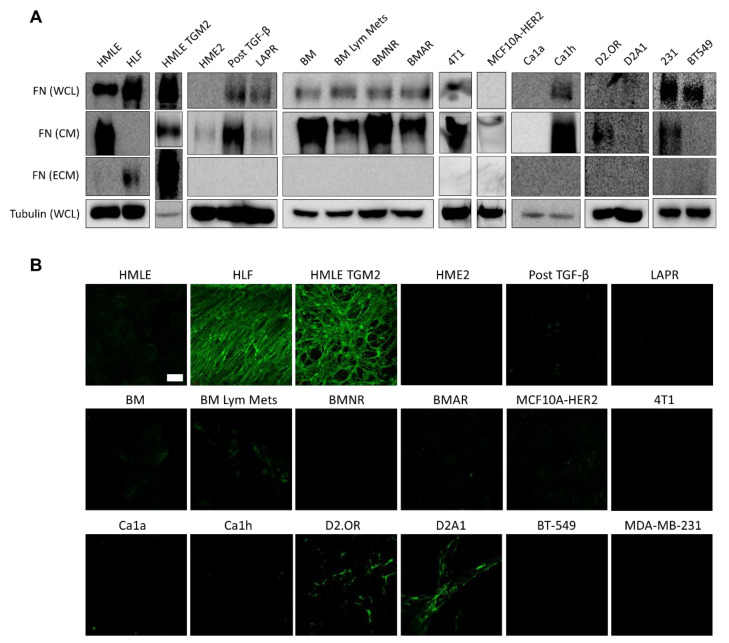
(**A**) Immunoblotting of the whole cell lysate (WCL) after trypsinization, conditioned media (CM), and extracellular matrix (ECM) of the 15 breast cancer (BC) cell lines indicated after 72 h in culture. None of these lines could produce fibrillar FN as an ECM. However, intracellular FN and soluble FN released into the media were detected from the majority of BC lines. Human lung fibroblasts (HLF) and mammary epithelial cells overexpressing transglutaminase (HMLE-TGM2) were used as positive controls for matrix deposition. (**B**) Immunofluorescent staining for FN in decellularized monolayers, performed in duplicate, showed limited FN matrix production by the BC cell lines as compared to HLF and HMLE-TGM2 cells. Scale bar is 50 µm.

We performed immunoblotting of the whole cell lysate (WCL), conditioned media (CM), and ECM deposited by 15 different BC cell lines (Figure 2A). Human mammary epithelial cells (HMLE) and human lung fibroblasts (HLFs) were used as control cells. HMLE-TG2 cells overexpress transglutaminase 2 (TG2). TG2 is an enzyme that can catalyze protein crosslinking of various extracellular matrix proteins, including laminin, collagen, and FN. Crosslinking via TG2 is linked to fibrosis and cancer progression [25]. We have also recently shown that TG2 emerges in metastatic BC cells that have undergone induction and reversal of EMT and can enhance metastasis if overexpressed in primary tumor cells [14]. 

The fifteen BC cell lines included multiple subtypes, drug sensitivities, invasive potentials, and represented various stages of the metastatic cascade. Cells were grouped according to similar lineage for immunoblotting (Figure 2A). We first investigated a HER2-transformed progression series. We used HER2-transformed human mammary epithelial cells (HME2) that are capable of primary tumor formation but have no metastatic potential [14]. Within the progression series, we used a HME2 line that had undergone drug-induced EMT via acquired resistance to the EGFR/HER2 kinase inhibitor, Lapatinib (LAPR) [26]. Separately, epithelial-mesenchymal plasticity (EMP) was induced in the HME2 line via a 4-week treatment with TGF-β1 followed by a 2-week withdrawal to create the Post TGF-β line. This EMP induction was sufficient to induce metastasis upon mammary fat pad engraftment [27]. Subculture of the resulting bone metastases established the epithelial BM line. Re-engraftment into the mammary fat pad and subculture of the resulting metastases in the lymph nodes gave rise to the BM Lym Mets line. The BMNR and BMAR lines were established by prolonged treatment of the BM cells with the pan-ErbB inhibitors, Neratinib and Afatinib, respectively, resulting in acquired stable resistance to these compounds. 

MCF10A-HER2 cells are an MCF10A derivative line that overexpress HER2 and are considered premalignant [28]. The remaining cell lines were from triple negative breast cancers (TNBC). The MCF10CA1a (Ca1a) and MCF10Ca1h (Ca1h) cells are derived from the RAS-transformed MCF-10AT cells and represent epithelial and mesenchymal populations, respectively [6,29,30]. D2.OR and D2A1 are two isogenic murine lines derived from mammary tumors originating from the D2 hyperplastic alveolar nodule (HAN) line. D2.OR cells exhibit characteristics of tumor cell dormancy in vivo and in Matrigel culture assays, while the D2A1 cells do not enter dormancy in soft 3D matrices and rapidly produce pulmonary tumors in mice [31]. The MDA-MB-231 (231) cell line is a widely used, invasive, TNBC line that was isolated from a pleural effusion of a patient with invasive ductal carcinoma. It is a common model of late-stage BC. Conversely, the BT-549 cell line was obtained from the primary tumor of a patient with invasive ductal carcinoma, but is also classified as basal B and claudin-low. Lastly, the 4T1 cell line is one of four sublines derived from the 410.4 tumor, which was isolated from a spontaneous mammary tumor in an MMTV^+^ BALB/c mouse. It is highly tumorigenic and invasive, and efficiently metastasizes to multiple sites that are also common in human BC including the lungs, liver, brain, and bone [32,33,34,35,36,37].

**Figure 3:**
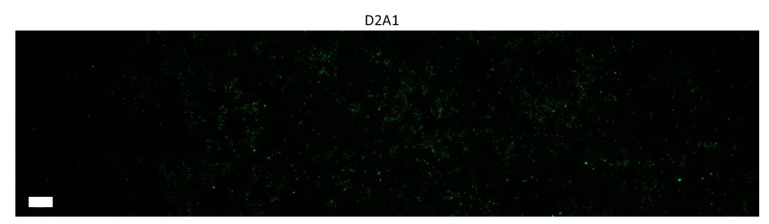
D2A1 cells produced the most robust FN fibrils seen by immunofluorescence of the BC cells tested. Representative image of an entire culture well indicated only sporadic deposition with no mature network after 72 h. Scale bar is 1 mm.

Our results indicated that a mature FN matrix was only formed from the fibroblast control cells and from the HMLE cells overexpressing TG2. While no BC cell line could produce a robust fibrillar FN matrix after 3 days, we observed sporadic deposition of FN, most notably in the D2 series, that was below the detectable level of immunoblotting (Figure 2B and Figure 3). The majority of BC cell lines (10/15) expressed FN in their whole cell lysate (Figure 2A, Figures S2 and S6–S8). Intracellular FN expression was not diminished after acquired drug resistance (BMNR and BMAR lines) and was enhanced after EMT as observed in the LAPR and post TGF-β cells compared to the HME2 parental line. Similarly, the majority of BC cells were able to secrete soluble FN into the media (12/15) (Figure 2A). The extracellular FN deposition was verified by immunostaining of decellularized cell layers. The majority of slides were completely negative, with a minority showing spots of fibrillar FN (Figure 2B). This staining confirms that BC cells can release soluble FN, but these cells do not readily organize a robust FN matrix. This result was irrespective of ascorbic acid addition to the media (Figure S2).

### 2.3. Fibroblasts Organize Exogenous Fibronectin into a Fibrillar Network

**Figure 4:**
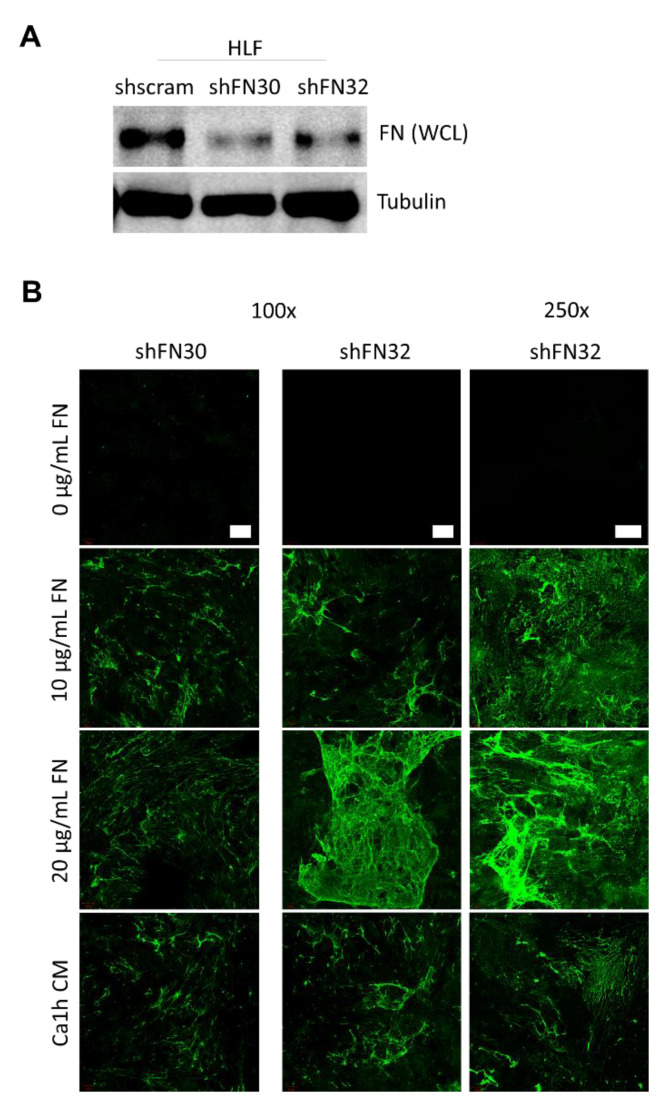
(**A**) FN expression was depleted in HLFs through lentiviral-mediated transduction of two distinct shRNAs. (**B**) Representative FN matrix visualization from decellularized FN-depleted HLF monolayers after 72 h in serum-free culture with 0–20 µg/mL exogenous FN, performed in duplicate. FN-depleted HLF cells could not produce fibrillar FN, but could organize exogenous FN into a matrix, including soluble FN produced by Ca1h BC cells. 100× scale bar is 100 µm. 250× scale bar is 50 µm.

Next, we explored the relationship between soluble FN produced by tumor cells and fibroblast matrix deposition. To this end, we depleted FN production in HLF cells through lentiviral-mediated transduction using two distinct shRNAs (shFN30 and shFN32). Depletion of intracellular levels of FN was verified by immunoblotting of whole cell lysates (Figure 4A and Figure S8). Immunostaining of decellularized slides indicated that no FN matrix was produced when the FN-depleted cells were cultured in standard serum-free media (Figure 4B). However, when soluble FN was added (10 µg/mL and 20 µg/mL), a fibrillar FN matrix was established that was indistinguishable from the matrix produced by wild-type HLF cells (Figure 4B and Figure 2B). In addition, when FN-depleted HLF cells were cultured in serum-free media conditioned by Ca1h cells for 72 h, a FN matrix was also established (Figure 4B). 

**Figure 5:**
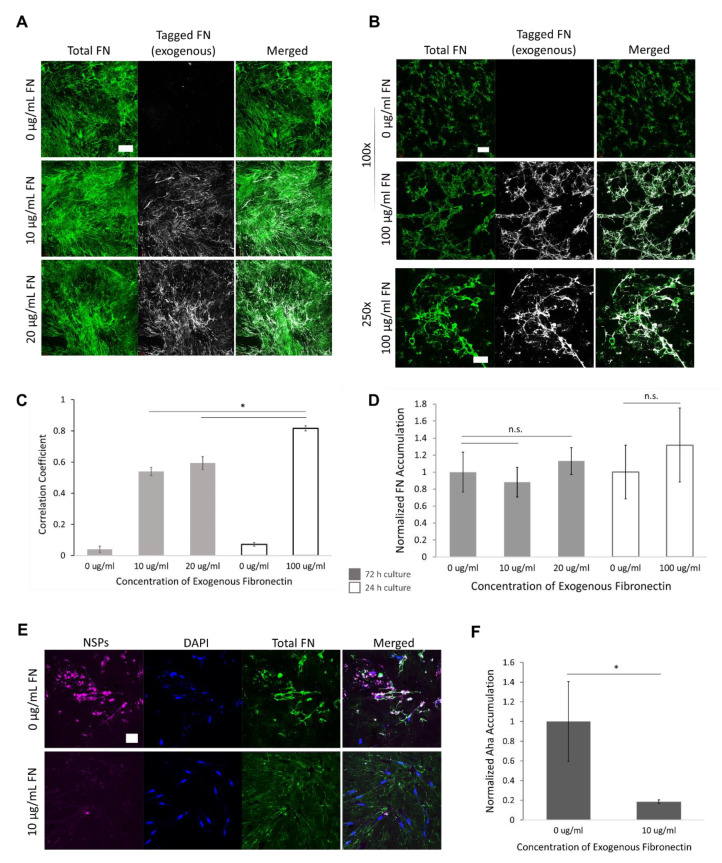
(**A**) Representative FN matrix visualization from decellularized wild-type HLF monolayers after 72 h in serum-free culture with 0–20 µg/mL exogenous FN, performed in duplicate. Exogenous FN is fluorescently labeled before addition into the culture media. Wild-type HLFs organize exogenous FN after 72 h which is indistinguishable from endogenous fibrillar FN. Scale bar is 50 µm (**B**) When culturing time is reduced from 72 to 24 h under high dosage of soluble FN, the resultant ECM is almost entirely from exogenous origins. 100× scalebar is 100 µm. 250× scalebar is 50 µm. (**C**) 100 ug/mL exogenous FN conditions cultured for 24 h resulted in a significantly higher correlation of exogenous FN with total FN than did lower doses and longer culturing times. (**D**) Exogenous FN did not alter the amount of the fibrillar network for a given culturing length. (**E**) Wild-type HLFs were cultured with the Met analog, Aha, for 48 h to visualize newly synthesized proteins (NSPs), performed in duplicate. Scale bar is 50 µm. (**F**) HLFs given exogenous FN show significantly lower levels of Aha-labeled proteins after 48 h, verifying incorporation of exogenous FN into wild-type HLF-produced matrices. (* indicates *p* < 0.05, n.s.: no significant).

Next, we sought to establish whether wild-type HLFs would incorporate exogenous soluble FN into a fibrillar matrix or if these cells would selectively utilize endogenous FN. This was accomplished in two ways. First, exogenous soluble FN was fluorescently labeled and incorporated into the serum-free media. This was performed for 3-day (Figure 5A) and 1-day culturing periods (Figure 5B) at various exogenous FN concentrations. In all cases, the exogenous FN was incorporated into a larger endogenous FN matrix. In these regions of clustered ECM, there was no difference in the amount of FN deposited with or without the exogenous supply (Figure 5D). However, under the shorter culturing period and higher exogenous FN concentration (100 µg/mL), there was a significantly larger correlation coefficient. This Pearson’s correlation coefficient is a measure of how similar the two image channels are, from 0 to 1, with 1 occurring if they are identical. The significantly larger value in the shorter culturing condition indicates that a larger percentage of the total FN matrix came from exogenous sources (Figure 5C). 

To verify these results, we used the non-canonical amino acid, azidohomoalanine (Aha), to label the proteins produced by wild-type HLF cells. Aha is a methionine (Met) analog that has a bio-orthogonal handle (azide) and is incorporated into newly synthesized proteins (NSPs) using the native translational machinery of the cell [38]. The NSPs can be visualized by using an alkyne-labeled fluorophore that is clicked to Aha via azide-alkyne cycloaddition [38,39]. As before, HLFs deposited a FN matrix after 48 h with or without the addition of 10 ug/mL soluble FN in the media. However, there was significantly less accumulation of Aha-labeled proteins in cultures given exogenous FN (Figure 5E,F). This confirmed that HLFs utilized exogenous and endogenous FN to form one indistinguishable FN matrix. 

### 2.4. Epithelial to Mesenchymal Plasticity Enhanced Fibronectin Accumulation in Paracrine Signaling

We next evaluated FN matrix deposition from HLFs after 48 h of exposure to CM from BC cells to mimic soluble factor conditioning via paracrine signaling. To delineate the effect of BC cell phenotype on HLF conditioning, we used media from the Ca1a cells, Ca1h cells, and Ca1h cells that had been specifically depleted of FN expression (Ca1h shFN30). Finally, to investigate the role of tumor heterogeneity on matrix production, co-cultures consisting of 50:50 Ca1a:Ca1h cells and Ca1a:Ca1h shFN30 cells were also utilized. 

**Figure 6:**
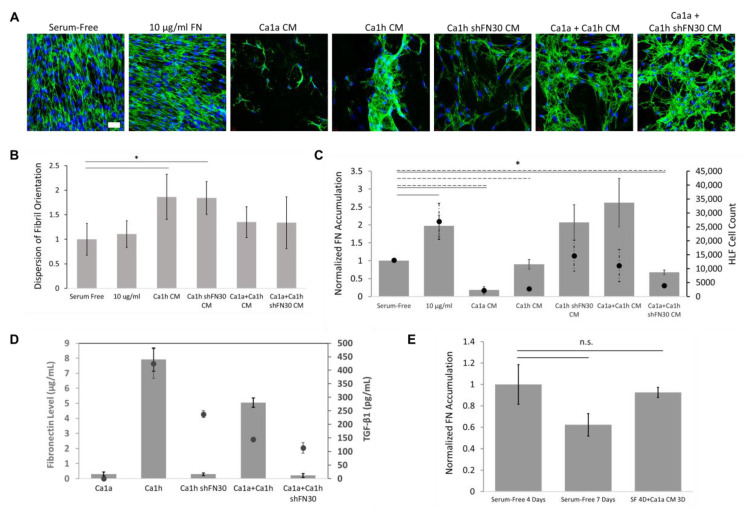
(**A**) BC-conditioned media affected the amount and orientation of fibrillar FN deposited by HLFs after 48 h (*n* = 4). Scale bar is 50 µm. (**B**) HLF cells deposited aligned FN fibrils in control samples, which was not disrupted by exogenous FN incorporation, but became more random in the presence of BC CM. Alignment was quantified from at least 3 images of high FN accumulation for each condition. (**C**) Exogenous FN resulted in higher total accumulation, while Ca1a and Ca1a:Ca1h shFN30 CM cultures resulted in less total accumulation by HLFs. Accumulation (bar, solid) was quantified from whole-plate tilescans of duplicate experiments. Conditioning HLFs with Ca1a, Ca1h, and Ca1a:Ca1h shFN30 CMs resulted in significantly less HLFs after 48 h (scatter, dashed). (**D**) FN accumulation was not directly correlated with the level of soluble FN (bar) or TGF-β1 (scatter) in CM samples (*n* = 3, mean ± s.d.). (**E**) When HLFs were cultured for 4 days in serum-free media and 3 additional days in Ca1a CM, Ca1a CM did not result in a breakdown of the already-existing FN matrix. (* indicates *p* < 0.05, n.s.: no significant).

HLFs given serum-free media produced a mature interconnected FN matrix within 48 h (Figure 6A). However, the network of matrix proteins was not confluent in the entire culture area (Figure S3). When given 10 µg/mL of soluble FN, significantly more FN matrix was present across the entire culture area (Figure 6C). Here, and in all subsequent experiments, FN accumulation was quantified from tilescan images of the full culture dish (Figure S3) for unbiased measurements. We analyzed the alignment of the FN fibrils by fitting a gaussian curve to the directionality distribution. The spread of the gaussian curve indicates the dispersion of fibril orientation where less dispersion correlates with a more aligned network of fibrils (Figure S4). At least three regions, approximately 0.7 mm^2^ each, of highest FN accumulation were analyzed per condition. Therefore, in the Ca1a CM cultures, which did not have regions of high FN accumulation, the dispersion was not quantified. We observed that serum-free cultured HLFs produced highly-aligned matrices, and that the addition of soluble FN did not alter this architecture (Figure 6A,B).

When HLF cells were cultured in CM from Ca1a cells, FN fibrillarization was diminished (Figure 6C). We then sought to investigate whether the Ca1a CM simply depressed FN matrix organization in HLFs, or if these factors would induce matrix breakdown (Figure 6E). HLFs were cultured in serum-free media for 4 or 7 days as controls. In the third group, HLFs were cultured in serum-free media for 4 days and then switched to Ca1a CM for 3 days. The HLFs were able to create a robust FN matrix within 4 days that covered the entire culture well (Figure S3). As such, there was no significant difference in the accumulation of FN between the 4 and 7 day culturing groups (Figure 6E). In cultures switched to Ca1a CM, there was also not a significant change in the amount of FN matrix (Figure 6E). These results suggest that the Ca1a CM inhibits accumulation of FN matrix deposition but does not alter the existing matrix. 

Interestingly, the depression of FN was not recapitulated by Ca1h shFN30 CM. HLFs cultured in Ca1h shFN30 CM produced on average more FN matrix, but the increase was not statistically significant compared to that of serum-free culturing (Figure 6C). Matrices from HLFs cultured in Ca1h and Ca1h shFN30 CM were also significantly less aligned than serum-free HLF matrices (Figure 6B). The global architecture of the Ca1h shFN30 CM group appeared similar to that of HLFs cultured with CM taken from a co-culture of Ca1a and Ca1h cells (Figure 6A and Figure S3). The level of accumulation seen from Ca1a:Ca1h CM culturing was the largest on average, producing more than either the Ca1a or Ca1h CMs individually (Figure 6C). 

The number of HLFs on the entire culture surface was quantified for each condition after 48 h. CM from Ca1a, Ca1h, and Ca1a:Ca1h shFN30 co-cultures resulted in statistically fewer HLFs (Figure 6D). Culturing HLFs in Ca1a CM also resulted in visually less healthy HLF cells (Figure 6A). There was partial agreement with the number of HLF cells and the overall FN accumulation per condition. Specifically, Ca1a and Ca1a:Ca1h shFN30 CM conditioning resulted in significantly lower FN accumulation and fewer HLF cells. However, the difference in FN accumulation cannot be explained solely by the promotion or inhibition of HLF population growth. For example, conditioning with Ca1h CM resulted in significantly fewer HLFs, without statistically affecting FN accumulation. Conversely, we observe no statistical difference in the number of HLFs after conditioning with Ca1h shFN30 and Ca1a:Ca1h CM, although both conditions resulted in an upward trend of FN accumulation (Figure 6D).

An ELISA was performed to quantify the amount of FN in each conditioned media sample (Figure 6D). As expected, the Ca1a and Ca1h shFN30 CM samples had very little soluble FN. The CM from Ca1h cells had a similar concentration of FN as the 10 ug/mL used for the exogenous group. Unsurprisingly, the Ca1a:Ca1h co-culture had less FN than the Ca1h media, as the number of each cell type seeded was halved to maintain the same total cell count. The concentration of FN was slightly (12%) higher than what would be calculated using these estimated cell numbers, despite the Ca1a cells having a faster proliferation rate. TGF-β1 has been shown to be a major regulator of FN production and a rapid inducer of fibrillogenesis [40,41,42]. We therefore investigated the concentration of TGF-β1 for each CM (Figure 6D). Our results show that media from native Ca1a epithelial BC cells have undetectable levels of TGF-β1 and media from Ca1h mesenchymal BC cells have >400 pg/mL of TGF-β1. Interestingly, the Ca1h shFN30 CM retains approximately half as much TGF-β1. In addition, while Ca1a:Ca1h CM retained approximately 62% soluble FN compared to the homogeneous controls, the co-cultured CM retained approximately 34% TGF-β1 concentration. 

### 2.5. A Mesenchymal Phenotype Enhanced Fibronectin Accumulation in Endocrine Signaling

**Figure 7:**
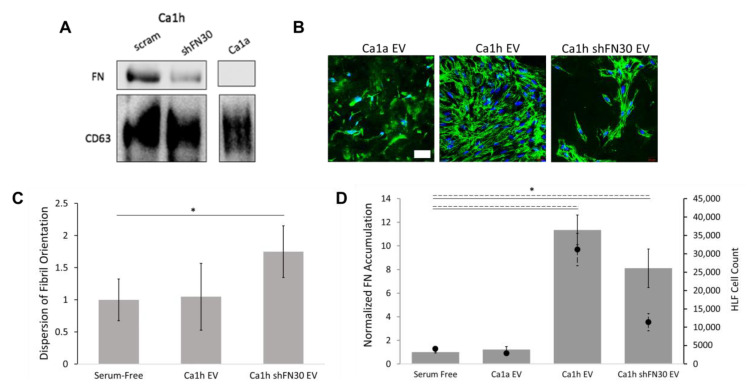
(**A**) FN levels in extracellular vesicle(EV)-enriched solutions from Ca1h, Ca1h shFN30, and Ca1a BC cells. (**B**) Representative images of FN matrices by HLFs after conditioning with EV-enriched solutions (*n* = 4). BC EVs affected the amount and orientation of fibrillar FN deposited by HLFs after 48 h. Scale bar is 50 µm. (**C**) Ca1h EV conditioning resulted in aligned FN fibrils by HLFs. Alignment was quantified from at least 3 images of high FN accumulation for each condition. (**D**) Ca1h and Ca1h shFN30 EVs resulted in significantly higher FN accumulation, while Ca1a EV conditioning produced no statistically significant effect. Accumulation (bar, solid) was quantified from whole-plate tilescans of duplicate experiments. Conditioning HLFs with EVs from Ca1h and Ca1h shFN30 cells resulted in significantly more HLFs after 48 h (scatter, dashed). (* indicates *p* < 0.05).

EVs released by BC cells at the primary tumor have been identified as major contributors of early metastatic niche conditioning [16,43,44]. We have previously demonstrated that FN is fibrillarized on the surface of EVs from some BC cells, and that EVs from these BC cells enhanced pulmonary tumor growth [14]. Here, EVs were enriched from Ca1a-, Ca1h-, and Ca1h shFN30-conditioned medias and added to HLF cultures. Immunoblotting indicated that Ca1h-derived EVs had FN, while Ca1h shFN30 and Ca1a EVs displayed little to no FN expression (Figure 7A). Of note, bicinchoninic acid (BCA) protein analyses indicated that EV-enriched preparations from Ca1h cultures had 6 times the protein and Ca1h shFN30 EV preparations had 3 times the protein as compared to Ca1a cultures.

Ca1a EVs did not improve matrix deposition by HLF cells. There was also no clear depression of total FN production due to Ca1a EVs as compared to the results obtained from unfractionated Ca1a CM, but no mature fibrillar architecture developed (Figure 7B,D). Therefore, dispersion was not quantified from Ca1a EV cultures. Ca1h EV conditioning significantly enhanced FN matrix deposition (Figure 7D and Figure S5). Interestingly, HLF cells conditioned with Ca1h EVs displayed an aligned architecture compared to what was seen from Ca1h CM (Figure 7B,C). Ca1h shFN30 EV conditioning similarly resulted in significantly more FN accumulation, but no mature architecture developed (Figure 7B,D). Conditioning HLFs with EV-enriched solutions from Ca1h and Ca1h shFN30 cells resulted in significantly more HLFs after 48 h, which corresponded directly with the increased total FN accumulation (Figure 7E).

**Figure 8:**
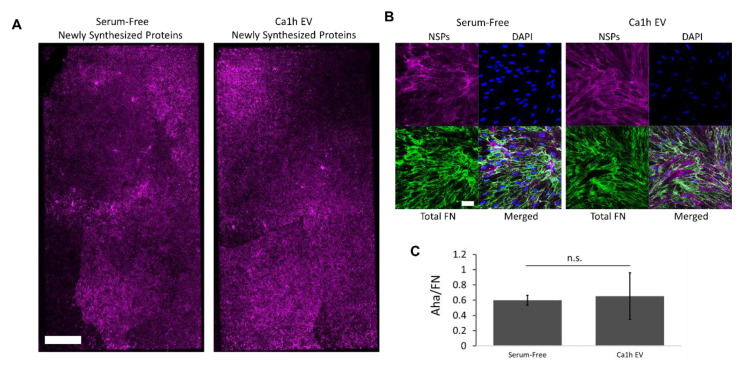
(**A**) Representative whole plate tilescan visualization of fluorescent labeling of Aha-tagged NSPs after 48 h, performed in duplicate. Scale bar is 1 mm. (**B**) Representative visualization of NSPs and FN staining of the ECM produced by wild-type HLFs after serum-free or Ca1h EV conditioning. Scale bar is 50 µm. (**C**). No difference was seen in the relative amount of Aha incorporation between control- and Ca1h EV-conditioned HLFs, indicating that all deposited FN was from endogenous origins.

When Aha was added to control HLF cells conditioned with Ca1h EVs, the bulk of the deposited FN matrix was shown to be NSPs (Figure 8A,B). There was no significant difference between the ratio of Aha/FN signal between control and Ca1h-EV-conditioned HLF cells (Figure 8C). The increase in FN accumulation was therefore not from HLFs directly utilizing the FN associated with the Ca1h EVs. 

## 3. Discussion

Work from our lab and others has demonstrated that the accumulation of extracellular FN is an indication of advancing BC disease [6,11,12,14,45]. Here, we investigated FN matrix formation in the primary tumor and metastatic lungs in vivo. We then systematically analyzed the intracellular, soluble, and extracellular levels of FN derived from BC cells. This approach revealed limited fibronectin deposition by any BC cell. We therefore investigated changes in fibrillar FN formation when fibroblasts were exposed to varied BC media fractions and demonstrated that matrix formation by fibroblasts can be influenced by BC cells. 

Using cleared primary 4T1 tumors, we observed mostly intracellular FN in the tumors within the first 10 d. However, by 15 d, we began to see the development of a robust matrix, and by 25 d we observe aligned tracks of FN that indicate potential routes of dissemination (Figure S1). Within the lungs, we observed a transient increase in FN expression from days 10–15 and a steady increase in Ki-67 staining beginning on day 15. These occurred before overt metastasis was seen, beginning on day 20, and before the formation of aligned FN tracks (Figure 1 and Figure S1). Due to the lack of a bioluminescent signal at earlier time points where Ki-67 staining was observed and the location of these proliferating cells throughout the lungs rather than in micrometastatic foci, these appear to be resident stromal cells. Similar observations in the lungs have been made by Kaplan et al., using the Lewis lung carcinoma model [11,45]. They reported an increase in FN in the premetastatic lungs from days 3–14 after tumor cell injection in mice. They also noted an increase in proliferation by resident fibroblast-like stromal cells in the lungs due to signaling from the primary tumor [45]. These results suggest that fibrillar FN must be down- and upregulated in target organs over time to facilitate the development of macrometastases.

By comparing non-transformed and tumorigenic epithelial cell lines, loss of intracellular FN expression has been previously correlated with malignant transformation [40]. A loss of FN in the WCL was seen when comparing the HMLE to the HER2 transformed HME2 cell lines. Furthermore, epithelial tumor cells, overall, displayed no intracellular FN, including the Ca1a cells (Figures S2 and S7). However, induction of EMT enhanced intracellular FN levels [4,6]. While the mesenchymal phenotype is often associated with increased invasion and resistance to anoikis, macrometastases were not detected from homogenous Ca1h tumors [6,46]. Research on early disseminated cancer cells and the latent reactivation suggests that while EMT or partial-EMT enables dissemination, cellular plasticity is required for these cells to enter a pro-growth phase and form clinically-detectable macrometastases [28,47]. In early disease progression, we have also shown that promoting EMP through the induction of a transient mesenchymal phenotype transforms a non-metastatic tumorigenic cell line into an effective metastasizer [14]. It is thus apparent that intracellular and autocrine FN expression is intimately tied with EMT and mesenchymal-epithelial transition (MET) status and requires transient expression at different stages of the metastatic cascade. Interestingly, we observed that BC cells readily secrete FN, as evidenced by its presence in the CM in 12/15 BC cell lines investigated. However, despite producing significant levels of FN, these cells did not readily form a mature fibrillar network (Figure 2).

Therefore, we investigated if fibroblasts could produce a fibrillar FN matrix using FN produced by cancer cells. We observed that fibroblasts do assemble an ECM network using exogenously supplied proteins, and the matrix network properties are dependent on the source of the FN (Figure 4, Figure 5 and Figure 6). Past work indicates that cancer-associated fibroblasts are a major effector of ECM production and remodeling in the tumor microenvironment [7]. Fibroblasts, often resident stromal fibroblasts, transition slowly into cancer associated fibroblasts via several mechanisms, including contact signals with BC cells, physiological stress, and soluble factors from the tumor [48]. Here, we investigated FN accumulation and alignment from fibroblasts conditioned by BC cell CM or EVs as a mimic of paracrine and endocrine signaling, respectively. 

For HLFs conditioned with BC CM, cultures with phenotypic heterogeneity resulted in the most FN accumulation, while epithelial-only CMs resulted in significantly lower accumulation compared to serum-free controls (Figure 6). Due to the association of fibrillar FN with the mesenchymal phenotype, it is unsurprising that HLFs conditioned with media from epithelial Ca1a cells experienced diminished FN matrix deposition compared to the control [4,49]. In addition, although late metastases are characterized by a stiffened ECM, stiffening too early at a metastatic site has been shown to push disseminated cancer cells into a dormant phenotype due to stress signals [13]. Therefore, FN matrix inhibition may be required for disseminated cancer cells to complete MET and form macrometastases [14,45]. Interestingly, although the FN expression was transient, the lung tissue density remained significantly higher in 15, 20, and 25 d mice as compared to wild-type mice (Figure 1). A growing number of proteins have been shown to depend on FN in order to be incorporated into the ECM, including collagens, fibrillins, and tenascin-C. Of particular note, FN and type I collagen have been co-localized in the secretory pathway of fibroblasts, and collagen fibrils do not accumulate in the absence of FN [16,19]. An increase in FN fibrils may act as an initial ECM foundation in the premetastatic niche, which is then replaced with an abundance of collagen I. 

Given the suppression of FN by epithelial BC cells, is it interesting that Ca1a:Ca1h co-cultured CM produced a more robust FN matrix than homogenous Ca1h CM (Figure 6). We have previously highlighted the synergistic dynamic of a heterogenous tumor, demonstrating that a Ca1a:Ca1h co-culture increased metastatic efficiency in vivo and increased survival in nutrient-starved in vitro collagen cultures compared to homogenous cultures [6,50]. The presence of both fractions likely leads to paracrine signaling that promotes overall EMP for the cell types. In addition, the presence of some post-EMT cells in the primary tumor may act as a spark that initiates FN accumulation as the environment transitions from pro-growth to pro-invasion. These findings also indicate why Ca1h shFN30 CM would produce a robust matrix similar to that of Ca1a:Ca1h CM. Our previous results demonstrated that depleting FN in Ca1h cells led to a mixed phenotype population, where both E-Cadherin and vimentin were expressed [6]. 

Importantly, conditioning HLFs with EV-enriched solutions to model formation of premetastatic niches via endocrine signaling indicated a different relationship between BC cell phenotype and FN accumulation. Our results suggest that the mesenchymal BC cells in the primary tumor most dramatically influence FN accumulation at metastatic niches (Figure 7), while EMP heterogeneity may facilitate more FN accumulation at the primary tumor (Figure 6). Through the use of noncanonical amino acid labeling we found that the bulk of the matrix accumulation induced by EVs occurs through NSPs by fibroblasts (Figure 8). Although the fibrillar FN associated with EVs is not directly incorporated into the ECM, FN present on the surface of the EVs may influence the rate of delivery to the secondary organ and increase the rate of EV consumption by HLFs. Others have similarly theorized that FN in the premetastatic niche increases the adhesion of exosomes and facilitates the colonization of circulating tumor cells [16]. 

In addition, modeling premetastatic niche formation using Ca1h EVs resulted in normal aligned FN matrices (Figure 7). In contrast, all of the BC CM groups resulted in matrix formation where the FN fibrils were no longer highly aligned. They instead clustered into pockets of FN, which then potentially interconnected in a more webbed pattern (Figure 6). This aberrant architecture matches what has been reported in the literature at the primary tumor [9]. Specifically, this radial fibril orientation is associated with primary tumor invasion and is not seen in metastatic niches [8,9,51]. Furthermore, previous research has associated several adhesion molecules on cancer-associated fibroblasts, including syndecan-1 and DDR2, with the regulation of matrix organization and stiffness in BC and should be investigated on the HLFs conditioned via CM or EV-enriched solutions [7,8]. Future studies will focus on an in-depth analysis of the HLFs after BC CM and EV conditioning.

Overall, we demonstrated through immunoblotting and fluorescent staining that BC cells themselves were not readily capable of completing the cell-mediated process of FN matrix deposition. Because the Ca1a:Ca1h CM and Ca1h shFN30 CM samples contained vastly different concentrations of TGF-β1 and FN, there are clearly additional soluble factors that influence FN matrix accumulation and architecture in primary tumor niches. One such factor may be TG2, which was shown in our previous work to be upregulated in cells with EMP and here to promote FN matrix formation from healthy mammary cells [14]. Previous literature has also highlighted lysyl oxidase (LOX) and fibroblast activation protein (FAP) as potential proteins of interest, which were beyond the scope of this work [52]. We note that the CM was collected from BC cultures without matrix or other cell types present. It is also likely that incorporating these would alter the factors released by the BC cells. For example, if Ca1a cells were directly seeded onto HLF cultures after FN matrix formation, an increase in matrix metalloproteinase (MMP) release may occur and result in matrix breakdown, which was not seen here [52]. While this work focused on FN accumulation directly from or due to BC cells, there is growing evidence that the stromal cells of the primary tumor microenvironment, such as transitioned cancer-associated fibroblasts, also have a substantial role in premetastatic niche formation [53,54]. The work presented here can be further expanded through detailed co-culturing of phenotypically static or plastic BC cells with tumor-associated stromal cells and analysis of the resulting matrices. 

## 4. Materials and Methods 

### 4.1. In Vivo Assays

All in vivo assays were conducted under IACUC (Institutional Animal Care and Use Committee) approval from the PACUC (Purdue Animal Care and Use Committee), protocol 1310000978A010. Luciferase expressing 4T1 cells were resuspended in phosphate buffered saline (PBS) (50 µL) and orthotopically engrafted onto the second mammary fat pad of 4 week old Balb/c mice (2.5 × 10^4^ cells/mouse) (Jackson Labs, Bar Harbor, ME, USA). Primary tumor growth and metastasis development were assessed via weekly bioluminescent imaging using the Advanced Molecular Imager (AMI) (Spectral Instruments, Tucson, AZ, USA). Based on previously established variability for bioluminescent imaging, 3 mice per group was chosen to adequately power our experiments to 0.80, with an α of 0.05. Mice were euthanized via CO_2_ inhalation and the lungs and primary tumors were immediately extracted and placed in 10% formalin for 24 h. One lobe of each lung and half of each primary tumor was then dehydrated in 70% ethanol. Following histological sectioning, 5 µm sections were stained using hematoxylin and eosin for visualization of tissue density and formation of pulmonary metastatic nodules.

Immunohistochemical analyses of formalin fixed paraffin embedded tissue sections from 4T1 pulmonary tumors were conducted by deparaffinization in xylene, rehydration, and antigen retrieval using 10 mM sodium citrate (pH 6.0) under pressurized boiling. After inactivation of endogenous peroxidases in 3% H_2_O_2_, primary antibodies specific to Ki-67 (BD biosciences, Cat#556003, 1:100 dilution) or FN (BD biosciences, Cat#610077, 1:1000 dilution) were added to serial sections and incubated overnight. Protein-specific staining was detected through the use of appropriate biotinylated secondary antibodies in conjunction with ABC reagent (Vector, Burlingame, CA, USA). These sections were counterstained with hematoxylin, dehydrated, and mounted. 

### 4.2. Tissue Clearing

After fixation in 10% formalin, one lobe of each lung and half of each primary tumor underwent tissue clearing following the X-CLARITY Systems protocol (Logos Biosystems, Annandale, VA, USA) [55]. Briefly, samples were submerged for 24 h at 4 °C in hydrogel initiator solution (1 part 25% (*w/v*) X-CLARITY Polymerization Initiator with 100 parts X-CLARITY Hydrogel Solution) (Cat#C1310X). Samples were then polymerized with the X-CLARITY Polymerization System at 37 °C for 3 h at 90 kPa and rinsed with PBS. Samples were put in the X-CLARITY Tissue Clearing System II and submerged with X-CLARITY Electrophoretic Tissue Clearing Solution (Cat#C13001) at 1.2A, 37 °C, with a pump rate of 60 rpm, until clear (approximately 16–18 h, but tissue-size dependent). 

Cleared samples were immunolabeled using the c-PRESTO (centrifugal pressure related efficient and stable transfer of macromolecules into organs) technique [55,56]. Briefly, samples were blocked with 6% bovine serum albumin and 0.1% TritonX-100 for 24 h and stained with FN (Sigma, Cat#3648) for 2 h at 4000 rpm and DAPI. Samples are placed in X-CLARITY mounting solution (Cat#C13101) for 1–2 h before imaging. 

### 4.3. Reagents

Ca1a and Ca1h cells were kindly provided by Dr. Fred Miller (Wayne State University, Detroit, MI, USA). MCF10A-HER2 cells were kindly provided by Dr. Julio Aguirre-Ghiso (Icahn School of Medicine at Mount Sinai, New York, NY, USA) [28]. The murine metastatic 4T1 cells were constructed to stably express firefly luciferase via transfection under Zeocin selection [57]. The HME2, Post TGF-β, LAPR, and the BM series of cells were previously established and described [14,27]. 

The majority of cell lines were cultured in DMEM/High Glucose media with 10% fetal bovine serum and 1% penicillin/streptomycin. The HER2+ BC cell lines (HMLE E2, HMLE Post TGF-β, LAPR, BM, BM Lym Mets, BMNR, and BMAR) were additionally cultured with 1% insulin. The HLFs were cultured in DMEM/High Glucose media with 10% fetal bovine serum and 1% penicillin/streptomycin or in fibroblast low-serum growth media (ATCC). All serum-free media was the standard media used without the fetal bovine serum.

### 4.4. Generation of HLF shFN30/32 Cell Line

Primary human pulmonary fibroblasts were obtained from ATCC and cultured in the recommended fibroblast basal media supplemented with fibroblast growth factor low serum kit (ATCC). Depletion of FN was achieved through lentiviral-mediated transduction of TRCN0000064830, TRCN0000064832, or a nontargeting, scrambled control shRNA, and stable expression was selected for using puromycin (GE Dharmacon, Lafayette, CO, USA). 

### 4.5. Generation of HMLE-TGM2 Cell Line

HMLE cells were kindly provided by Sendurai Mani (MD Anderson Cancer Center). These cells were cultured in HMLE media as previously described [17]. Overexpression of TG2 was achieved through lentiviral-mediated transduction of pLV (VectorBuilder, Santa Clara, CA, USA) encoding full length human TGM2 or GFP as a control. Stable genomic integration of constructs was selected for using puromycin. HMLE-TGM2 cells were cultured in full growth media for 6 weeks, which resulted in the mesenchymal HMLE-TGM2 cell line utilized for the studies. 

### 4.6. Generation of HME2-BMNR and HME2-BMAR Cell Lines

HME2-BM cells were treated with 100 nM neratinib or afatinib for 8 weeks to obtain HME2-BM neratinib-resistant and HME2-BM afatinib-resistant cell lines (HME2-BMNR and HME2-BMAR).

### 4.7. Decellularization

Cells in chamber slides or 6 well plates were decellularized before immunoblotting or immunostaining for FN (Sigma, St. Louis, MI, USA, Cat#3648, 1:200 dilution) when indicated as previously described [6,58]. Starting with live cells, cells were washed with PBS then deionized water. The samples were immersed in a 0.1% TX100 with 1.5 M KCL in 50 mM Tris buffer solution at 4 °C for 2 h on a slow-moving shaker. Samples were washed in 10 mM Tris buffer and then deionized water for 1 h each on a slow-moving shaker. 

### 4.8. Fluorescent Imaging

For immunofluorescence, plates were fixed in 4% paraformaldehyde and stained with polyclonal FN antibody (Sigma, Cat#3648, 1:200 dilution). If not decellularized, cells were also stained with DAPI, but were never permeabilized during staining. In studies involving labeling with Aha, cells were cultured for two days in glutamine-, methionine- and cystine-free high-glucose DMEM (Gibco, Waltham, MA, USA) containing 1% penicillin/streptomycin (Gibco), 1% glutagro (Corning, Corning, NY, USA), 0.2 mM cystine (Sigma), and 40 µg/mL of either Aha (Click Chemistry Tools, Scottsdale, AZ, USA) or Met (Sigma) as a control. For nascent protein visualization, cells were incubated in fresh media without Aha or methionine, but with 30 μM Alexa Fluor DBCO-647 (Click Chemistry Tools) for 30 min at 37  °C. Following incubation, cells were washed twice with PBS and fixed in 4% paraformaldehyde for 30 min at room temperature [38,39]. 

### 4.9. Immunoblot Analysis

For immunoblot analyses, cells were lysed using a modified RIPA lysis buffer containing 50 mM Tris, 150 mM NaCl, 0.25% Sodium Deoxycholate, 1.0% NP40, 0.1% SDS, protease inhibitor cocktail, 10 mM activated sodium ortho-vanadate, 40 mM β-glycerolphosphate, and 20 mM sodium fluoride. These lysates were separated by reducing SDS PAGE and probed for FN (BD biosciences, Cat#610077, 1:250 dilution) or β-tubulin (DSHB, Iowa City, IA, USA, Cat#E7). Where indicated, cellular monolayers were removed through incubation with 0.4% triton, 1.5 NaCl, 50 mM, Tris pH 8, and 50 mM EDTA for 48 h in 4 °C, washed with water, incubated with 0.5% sodium deoxycholate at 25 °C, and finally incubated with PBS (+MgCl_2_) for 1 h. Secreted proteins were precipitated from serum-free media by incubating 6 volumes of sample with 1 volume of 50% trichloroacetic acid and 1 volume of 0.1% sodium deoxycholate on ice for 30 min. Protein was precipitated via centrifugation and the pellet was twice washed with acetone and finally boiled at 95 °C with 500 µL of 4× laemmli buffer, separated by reducing SDS PAGE, and probed for FN (BD biosciences, San Jose, CA, USA, Cat#610077, 1:250 dilution). 

### 4.10. Conditioned Media Assays

To generate the conditioned media, cells were seeded at a density of 6 × 10^5^ cells per well in a 6-well plate (Corning Cell Culture plate). After 24 h, with the cells at around 80% confluent, the medium was switched to serum-free media (DMEM/High Glucose with 1% penicillin/streptomycin). After 30 h, the media was collected, filtered using a 0.2 um filter, and stored at −80 °C. After media collection, the cells were washed with PBS, trypsinized, and counted. Then, 50,000 HLFs were cultured in each well of a 4-well chamber slide in serum-free media or in the media collected from the BC cultures for 48 h. The amount of CM used was normalized to the same number of BC cells per condition. 

To assess the amount of FN found in the conditioned media samples, an ELISA was performed (Human Fibronectin ELISA kit; Abcam, Cambridge, UK). A filtered aliquot was thawed and used as the sample. The FN standard was reconstituted with the appropriate buffer, however, standards were made using serum-free media (DMEM/High Glucose with 1% penicillin/streptomycin) to better represent the sample conditions. The assay was performed following the protocol provided by the manufacturer. To evaluate the TGF-β1 content in the conditioned media samples, an ELISA was performed (TGF-β1 Human ELISA kit; Thermofisher, Waltham, MA, USA). Samples were not diluted 1:10 with Assay Buffer as stated in the protocol. Instead, 200 µl of thawed conditioned media was used. The assay was then performed following the protocol provided by the manufacturer.

The cell pellet was resuspended in Pierce RIPA buffer (1 mL per 5 × 10^6^ cells) (Thermo Scientific) and placed on a shaker on ice for 30 min. These samples were stored at −20 °C until use. The lysed cell samples were thawed and spun down at 14,000 g for 10 min. The supernatant was used for the BCA assay, in accordance with the protocol provided by the manufacturer (Micro BCA Protein Assay Kit; Thermo Scientific, Waltham, MA, USA).

### 4.11. Extracellular Vesicles Assays

EVs were enriched from BC CM as previously described from 2 T175, 5-layer flasks (Corning Cat#353144) after 72 h serum-free conditioning (~2 × 10^8^ BC cells per type). Briefly, CM samples underwent serial centrifugation from 300 to 2000× *g*, 0.22-µMpore size filtration, 3-KDa molecular weight cut-off concentration, and ultracentrifugation at 100,000 × g [14]. Pellets were additionally washed with PBS using ultracentrifugation. Pelleted EVs were resuspended in PBS and stored at −80°C. A portion of the solution was used for a BCA assay, using dilutions from 1:100–1:10,000 in a RIPA/PBS mixture, according to the manufacturer’s instructions (Micro BCA Protein Assay Kit; Thermo Scientific). Then, 50,000 HLFs were cultured in each well of a 4-well chamber slide in serum-free media. Each well was given 5 µg total protein of EV solution from one BC cell type and fixed after 48 h.

### 4.12. Statistical Analyses

Two-sided T tests were used where the data met the assumptions of these tests and the variance was similar between the two groups being compared. *p* values of <0.05 were considered significant. No exclusion criteria were utilized in these studies. All data are reported as the mean and sample standard deviation. 

## 5. Conclusions

Analysis of BC patient data has correlated FN matrix accumulation with the poorest survival outcomes irrespective of subtype [6]. Accumulation of FN has also been reported to predict where macrometastases will form and has been traced to resident stromal origins and primary tumor origins [12]. In this work, we have indicated that BC cells cannot produce a mature FN matrix regardless of subtype, EMT status, metastatic potential, or acquired chemotherapeutic resistance. We further demonstrated that FN production, release, and matrix organization involves separate mechanisms in both BC and fibroblast cells. Resident fibroblasts will incorporate both exogenous and endogenous soluble FN into a complete matrix and are heavily influenced by the soluble factors released by BC cells. Homogeneous epithelial BC cultures inhibit HLF FN matrix organization, while homogenous mesenchymal BC cultures do not. However, cultures with EMP characteristics, such as Ca1a:Ca1h co-cultures and Ca1h shFN30 cultures result in the most robust matrices from HLF cells, which is not directly correlated with the amount of soluble FN or TGF-β1 in the media. Lastly, endocrine conditioning of fibroblasts via BC EVs in early metastatic niche development seems dependent on a stable mesenchymal phenotype, which resulted in a significantly greater FN matrix. Despite this, the FN in and on the extracellular vesicles did not directly contribute to this matrix accumulation. In conclusion, we have demonstrated site-dependent and phenotype-dependent fibroblast conditioning, resulting in varied FN matrix accumulation as it relates to tumor microenvironment changes throughout primary tumor development, invasion, and macrometastasis. 

## Supplementary Materials

The following are available online at https://www.mdpi.com/2072-6694/12/5/1270/s1. Figure S1: (A) H&E of the primary tumor sections and (B) cleared whole tumors after 4T1 mammary fat pad injection show an increase in density of the tumor cells and an increase in extracellular matrix proteins over time. (A) Scale bar is 200 µm. (B) Scale bar is 50 µm, Figure S2: (A) Combined immunoblot of control and BC cells demonstrated an initial loss of autocrine FN after tumorigenesis, which was regained after EMT/EMP. Overall, Ca1h cells produced the most soluble FN and TG2-overexpressed HMLE cells produced the most autocrine and ECM FN. (B) Immunoblotting verification of WCL and soluble FN levels of conditioned media collected from Ca1a, Ca1h, Ca1a FN over-expressed (OE), and Ca1h shFN30 cells. Previously-transformed Ca1a FNOE cells did not retain FN expression during this culturing and were therefore not used in conditioning experiments. (C) The production of a mature network and (D) overall accumulation of fibrillar FN was not enhanced by the addition of ascorbic acid in HLF or HME2 cells. (C) Scale bar is 50 µm. (D) Scale bar is 1 mm, Figure S3: Representative tilescan of FN when HLFs were conditioned with (A) endogenous FN, BC CM, or (B) CM from BC co-cultures. (C) Representative tilescan of FN when partially cultured with Ca1a CM. Scale bar is 1 mm, Figure S4: Example directionality histogram for one image in the serum-free HLF group. The amount of fibrils is displayed per direction (180°), binned in 2° increments. The data is fit with a gaussian curve and the dispersion is the standard deviation of the gaussian function, Figure S5: Representative tilescan of FN when HLFs were conditioned with EVs from BC cells. Scale bar is 1 mm, Figure S6: Uncropped immunoblotting of each BC and control cell, grouped by similar lineage, with molecular weight markers and densitometry readings, Figure S7: Uncropped combined immunoblotting of the BC and control cells, with molecular weight markers and densitometry readings, Figure S8: Uncropped immunoblotting with molecular weight markers and densitometry readings for the (A) Ca1a, Ca1h, Ca1a FNOE, Ca1h shFN30 cells, and co-cultures, the (B) HLFs, and the (C) EVs from Ca1h, Ca1h shFN30, and Ca1a cells.

## References

[1] Siegel R.L., Miller K.D., Jemal A. (2019). Cancer statistics, 2019. CA Cancer J. Clin..

[2] Dillekås H., Rogers M.S., Straume O. (2019). Are 90% of deaths from cancer caused by metastases?. Cancer Med..

[3] Guan X. (2015). Cancer metastases: Challenges and opportunities. Acta Pharm. Sin. B.

[4] Insua-Rodríguez J., Oskarsson T. (2016). The extracellular matrix in breast cancer. Adv. Drug Deliv. Rev..

[5] Balanis N., Wendt M.K., Schiemann B.J., Wang Z., Schiemann W.P., Carlin C.R. (2013). Epithelial to mesenchymal transition promotes breast cancer progression via a fibronectin-dependent STAT3 signaling pathway. J. Biol. Chem..

[6] Shinde A., Libring S., Alpsoy A., Abdullah A., Schaber J.A., Solorio L., Wendt M.K. (2018). Autocrine Fibronectin Inhibits Breast Cancer Metastasis. Mol. Cancer Res..

[7] Bayer S.V., Grither W.R., Brenot A., Hwang P.Y., Barcus C.E., Ernst M., Pence P., Walter C., Pathak A., Longmore G.D. (2019). DDR2 controls breast tumor stiffness and metastasis by regulating integrin mediated mechanotransduction in CAFs. Elife.

[8] Yang N., Mosher R., Seo S., Beebe D., Friedl A. (2011). Syndecan-1 in breast cancer stroma fibroblasts regulates extracellular matrix fiber organization and carcinoma cell motility. Am. J. Pathol..

[9] Clark A.G., Vignjevic D.M. (2015). Modes of cancer cell invasion and the role of the microenvironment. Curr. Opin. Cell Biol..

[10] Medeiros B., Goodale D., Postenka C., Lowes L.E., Kiser P., Hearn S., Salmond N., Williams K.C., Allan A.L. (2020). Triple-Negative Primary Breast Tumors Induce Supportive Premetastatic Changes in the Extracellular Matrix and Soluble Components of the Lung Microenvironment. Cancers (Basel).

[11] Kaplan R.N., Rafii S., Lyden D. (2006). Preparing the “soil”: The premetastatic niche. Cancer Res..

[12] Sleeman J.P. (2012). The metastatic niche and stromal progression. Cancer Metastasis Rev..

[13] Libring S., Solorio L., Park K. (2020). 16 - Cancer mechanobiology: interaction of biomaterials with cancer cells. Biomaterials for Cancer Therapeutics.

[14] Shinde A., Paez J.S., Libring S., Hopkins K., Solorio L., Wendt M.K. (2020). Transglutaminase-2 facilitates extracellular vesicle-mediated establishment of the metastatic niche. Oncogenesis.

[15] Wortzel I., Dror S., Kenific C.M., Lyden D. (2019). Exosome-Mediated Metastasis: Communication from a Distance. Dev. Cell.

[16] Guo Y., Ji X., Liu J., Fan D., Zhou Q., Chen C., Wang W., Wang G., Wang H., Yuan W. (2019). Effects of exosomes on pre-metastatic niche formation in tumors. Mol. Cancer.

[17] Rackov G., Garcia-Romero N., Esteban-Rubio S., Carrión-Navarro J., Belda-Iniesta C., Ayuso-Sacido A. (2018). Vesicle-Mediated Control of Cell Function: The Role of Extracellular Matrix and Microenvironment. Front. Physiol..

[18] Akhtar M., Haider A., Rashid S., Al-Nabet A.D.M.H. (2019). Paget’s “Seed and Soil” Theory of Cancer Metastasis: An Idea Whose Time has Come. Adv. Anat. Pathol..

[19] Deep G., Jain A., Kumar A., Agarwal C., Kim S., Leevy W.M., Agarwal R. (2020). Exosomes secreted by prostate cancer cells under hypoxia promote matrix metalloproteinases activity at pre-metastatic niches. Mol. Carcinog..

[20] McKeown-Longo P.J., Mosher D.F. (1983). Binding of plasma fibronectin to cell layers of human skin fibroblasts. J. Cell Biol..

[21] Singh P., Carraher C., Schwarzbauer J.E. (2010). Assembly of fibronectin extracellular matrix. Annu. Rev. Cell Dev. Biol..

[22] Kadler K.E., Hill A., Canty-Laird E.G. (2008). Collagen fibrillogenesis: Fibronectin, integrins, and minor collagens as organizers and nucleators. Curr. Opin. Cell Biol..

[23] Jakhu H., Gill G., Singh A. (2018). Role of integrins in wound repair and its periodontal implications. J. Oral. Biol. Craniofac. Res..

[24] Erler J.T., Bennewith K.L., Cox T.R., Lang G., Bird D., Koong A., Le Q.T., Giaccia A.J. (2009). Hypoxia-induced lysyl oxidase is a critical mediator of bone marrow cell recruitment to form the premetastatic niche. Cancer Cell.

[25] Wang X., Yu Z., Zhou Q., Wu X., Chen X., Li J., Zhu Z., Liu B., Su L. (2016). Tissue transglutaminase-2 promotes gastric cancer progression via the ERK1/2 pathway. Oncotarget.

[26] Brown W.S., Akhand S.S., Wendt M.K. (2016). FGFR signaling maintains a drug persistent cell population following epithelial-mesenchymal transition. Oncotarget.

[27] Shinde A., Hardy S.D., Kim D., Akhand S.S., Jolly M.K., Wang W.H., Anderson J.C., Khodadadi R.B., Brown W.S., George J.T. (2019). Spleen Tyrosine Kinase-Mediated Autophagy Is Required for Epithelial-Mesenchymal Plasticity and Metastasis in Breast Cancer. Cancer Res..

[28] Harper K.L., Sosa M.S., Entenberg D., Hosseini H., Cheung J.F., Nobre R., Avivar-Valderas A., Nagi C., Girnius N., Davis R.J. (2016). Mechanism of early dissemination and metastasis in Her2(+) mammary cancer. Nature.

[29] Santner S.J., Dawson P.J., Tait L., Soule H.D., Eliason J., Mohamed A.N., Wolman S.R., Heppner G.H., Miller F.R. (2001). Malignant MCF10CA1 cell lines derived from premalignant human breast epithelial MCF10AT cells. Breast Cancer Res. Treat.

[30] Strickland L.B., Dawson P.J., Santner S.J., Miller F.R. (2000). Progression of premalignant MCF10AT generates heterogeneous malignant variants with characteristic histologic types and immunohistochemical markers. Breast Cancer Res. Treat.

[31] Morris V.L., Tuck A.B., Wilson S.M., Percy D., Chambers A.F. (1993). Tumor progression and metastasis in murine D2 hyperplastic alveolar nodule mammary tumor cell lines. Clin. Exp. Metastasis.

[32] Welsh J., Conn P.M. (2013). Chapter 40—Animal Models for Studying Prevention and Treatment of Breast Cancer. Animal Models for the Study of Human Disease.

[33] Dai X., Cheng H., Bai Z., Li J. (2017). Breast Cancer Cell Line Classification and Its Relevance with Breast Tumor Subtyping. J. Cancer.

[34] Neve R.M., Chin K., Fridlyand J., Yeh J., Baehner F.L., Fevr T., Clark L., Bayani N., Coppe J.P., Tong F. (2006). A collection of breast cancer cell lines for the study of functionally distinct cancer subtypes. Cancer Cell.

[35] Chavez K.J., Garimella S.V., Lipkowitz S. (2010). Triple negative breast cancer cell lines: One tool in the search for better treatment of triple negative breast cancer. Breast Dis..

[36] Pulaski B.A., Ostrand-Rosenberg S. (2001). Mouse 4T1 breast tumor model. Curr. Protoc. Immunol..

[37] Tao K., Fang M., Alroy J., Sahagian G.G. (2008). Imagable 4T1 model for the study of late stage breast cancer. BMC Cancer.

[38] Saleh A.M., Wilding K.M., Calve S., Bundy B.C., Kinzer-Ursem T.L. (2019). Non-canonical amino acid labeling in proteomics and biotechnology. J. Biol. Eng..

[39] Calve S., Witten A.J., Ocken A.R., Kinzer-Ursem T.L. (2016). Incorporation of non-canonical amino acids into the developing murine proteome. Sci. Rep..

[40] Lin T.C., Yang C.H., Cheng L.H., Chang W.T., Lin Y.R., Cheng H.C. (2019). Fibronectin in Cancer: Friend or Foe. Cells.

[41] Peng F., Zhang B., Wu D., Ingram A.J., Gao B., Krepinsky J.C. (2008). TGFbeta-induced RhoA activation and fibronectin production in mesangial cells require caveolae. Am. J. Physiol. Renal. Physiol..

[42] Varadaraj A., Jenkins L.M., Singh P., Chanda A., Snider J., Lee N.Y., Amsalem-Zafran A.R., Ehrlich M., Henis Y.I., Mythreye K. (2017). TGF-β triggers rapid fibrillogenesis via a novel TβRII-dependent fibronectin-trafficking mechanism. Mol. Biol. Cell.

[43] Chin A.R., Wang S.E. (2016). Cancer-derived extracellular vesicles: The ‘soil conditioner’ in breast cancer metastasis?. Cancer Metastasis Rev..

[44] Tian W., Liu S., Li B. (2019). Potential Role of Exosomes in Cancer Metastasis. Biomed. Res. Int..

[45] Kaplan R.N., Riba R.D., Zacharoulis S., Bramley A.H., Vincent L., Costa C., MacDonald D.D., Jin D.K., Shido K., Kerns S.A. (2005). VEGFR1-positive haematopoietic bone marrow progenitors initiate the pre-metastatic niche. Nature.

[46] Cao Z., Livas T., Kyprianou N. (2016). Anoikis and EMT: Lethal “Liaisons” during Cancer Progression. Crit. Rev. Oncog..

[47] Weidenfeld K., Barkan D. (2018). EMT and Stemness in Tumor Dormancy and Outgrowth: Are They Intertwined Processes?. Front. Oncol..

[48] Sahai E., Astsaturov I., Cukierman E., DeNardo D.G., Egeblad M., Evans R.M., Fearon D., Greten F.R., Hingorani S.R., Hunter T. (2020). A framework for advancing our understanding of cancer-associated fibroblasts. Nat. Rev. Cancer.

[49] Ondeck M.G., Kumar A., Placone J.K., Plunkett C.M., Matte B.F., Wong K.C., Fattet L., Yang J., Engler A.J. (2019). Dynamically stiffened matrix promotes malignant transformation of mammary epithelial cells via collective mechanical signaling. Proc. Natl. Acad. Sci. USA.

[50] Jun B., Guo T., Libring S., Chanda M., Buno K., Paez J.S., Shinde A., Wendt M., Vlachos P., Solorio L. (2020). Fibronectin-Expressing Mesenchymal Tumor Cells Promote Breast Cancer Metastasis.

[51] Cox T.R., Erler J.T. (2011). Remodeling and homeostasis of the extracellular matrix: Implications for fibrotic diseases and cancer. Dis. Model. Mech..

[52] Wang K., Wu F., Seo B.R., Fischbach C., Chen W., Hsu L., Gourdon D. (2017). Breast cancer cells alter the dynamics of stromal fibronectin-collagen interactions. Matrix Biol..

[53] Kong J., Tian H., Zhang F., Zhang Z., Li J., Liu X., Li X., Liu J., Jin D., Yang X. (2019). Extracellular vesicles of carcinoma-associated fibroblasts creates a pre-metastatic niche in the lung through activating fibroblasts. Mol. Cancer.

[54] Psaila B., Lyden D. (2009). The metastatic niche: Adapting the foreign soil. Nat. Rev. Cancer.

[55] Du H., Hou P., Zhang W., Li Q. (2018). Advances in CLARITY-based tissue clearing and imaging. Exp. Ther. Med..

[56] Lee E., Sun W. (2016). ACT-PRESTO: Biological Tissue Clearing and Immunolabeling Methods for Volume Imaging. J. Vis. Exp..

[57] Wendt M.K., Schiemann W.P. (2009). Therapeutic targeting of the focal adhesion complex prevents oncogenic TGF-beta signaling and metastasis. Breast Cancer Res..

[58] Jordahl S., Solorio L., Neale D.B., McDermott S., Jordahl J.H., Fox A., Dunlay C., Xiao A., Brown M., Wicha M. (2019). Engineered Fibrillar Fibronectin Networks as Three-Dimensional Tissue Scaffolds. Adv. Mater..

