# Supplementary material for: The Dynamic Relationship of Breast Cancer Cells and Fibroblasts in Fibronectin Accumulation at Primary and Metastatic Tumor Sites
Source: Cancers (Basel). 2020 May 17;12(5):1270. doi: 10.3390/cancers12051270 (PMC7281295; doi:10.3390/cancers12051270)
Supplement: Supplementary file 1 [file cancers-12-01270-s001.pdf]

Supplementary Materials: The Dynamic Relationship of Breast Cancer Cells and Fibroblasts in Fibronectin Accumulation at Primary and Metastatic Tumor Sites

Sarah Libring, Aparna Shinde, Monica K. Chanda, Maryam Nuru, Heather George, Aya M. Saleh, Ammara Abdullah, Tamara L. Kinzer-Ursem, Sarah Calve, Michael K. Wendt and Luis Solorio

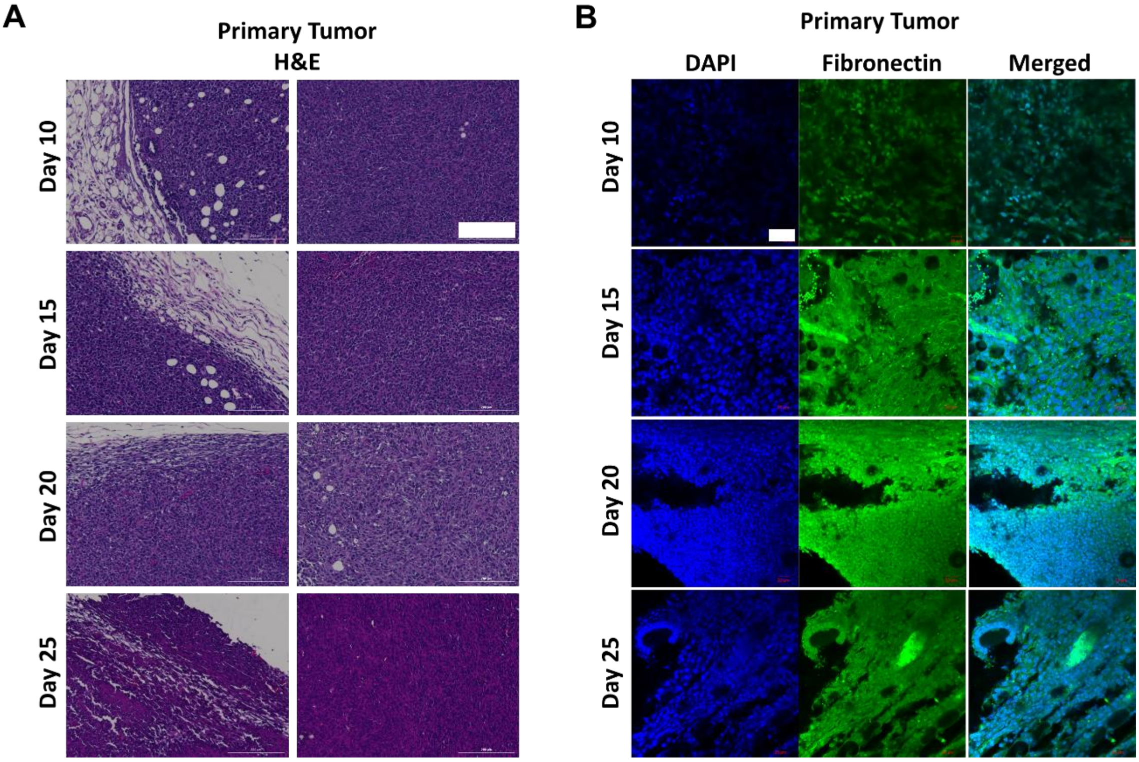

Figure S1. (**A**) H&E of the primary tumor sections and (**B**) cleared whole tumors after 4T1 mammary fat pad injection show an increase in density of the tumor cells and an increase in extracellular matrix proteins over time. (A) Scale bar is 200 μm. (B) Scale bar is 50 μm.

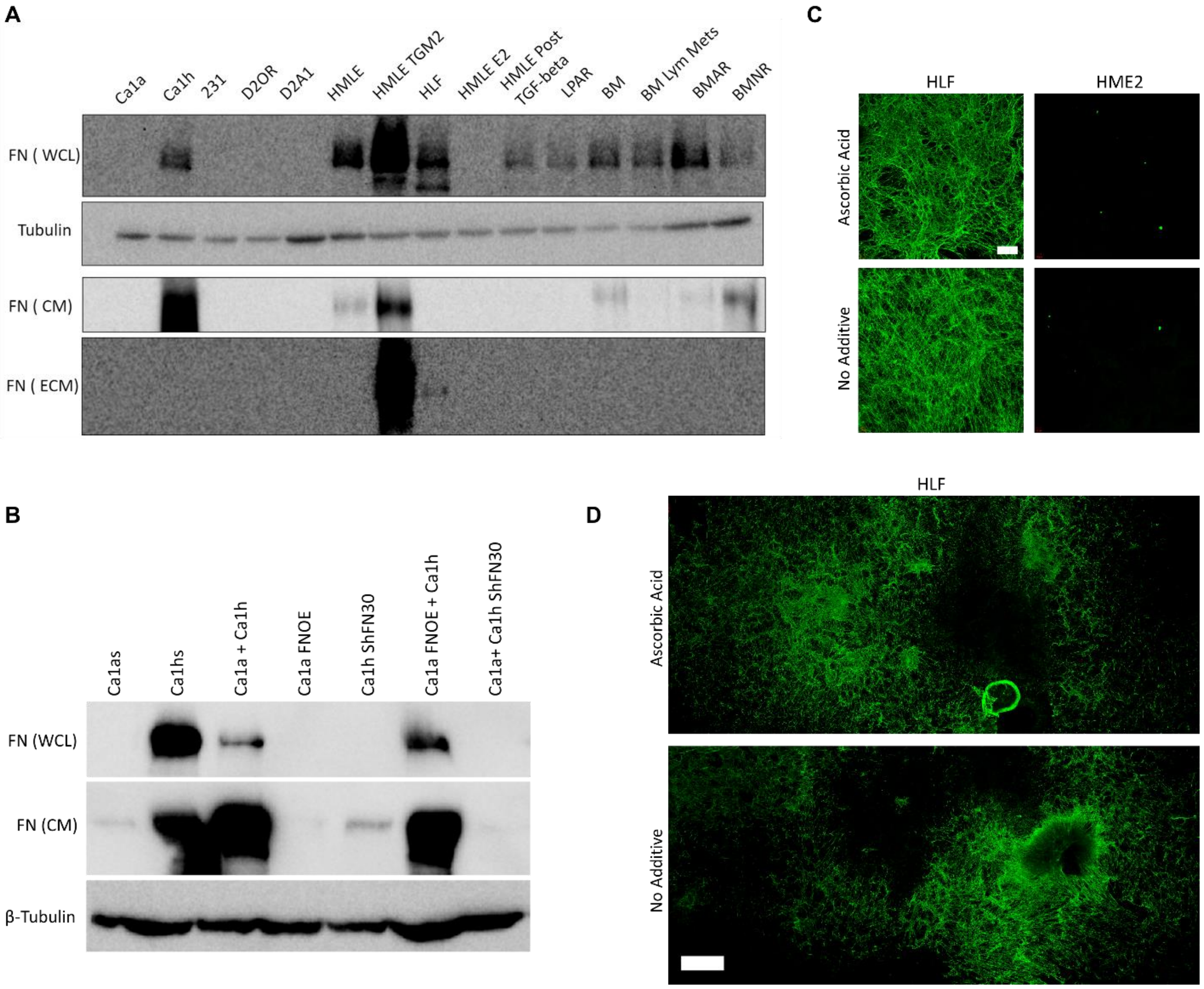

Figure S2. (**A**) Combined immunoblot of control and BC cells demonstrated an initial loss of autocrine FN after tumorigenesis, which was regained after EMT/EMP. Overall, Ca1h cells produced the most soluble FN and TG2-overexpressed HMLE cells produced the most autocrine and ECM FN. (**B**) Immunoblotting verification of WCL and soluble FN levels of conditioned media collected from Ca1a, Ca1h, Ca1a FN over-expressed (OE), and Ca1h shFN30 cells. Previously-transformed Ca1a FNOE cells did not retain FN expression during this culturing and were therefore not used in conditioning experiments. (**C**) The production of a mature network and (**D**) overall accumulation of fibrillar FN was not enhanced by the addition of ascorbic acid in HLF or HME2 cells. (C) Scale bar is 50 μm. (D) Scale bar is 1 mm.

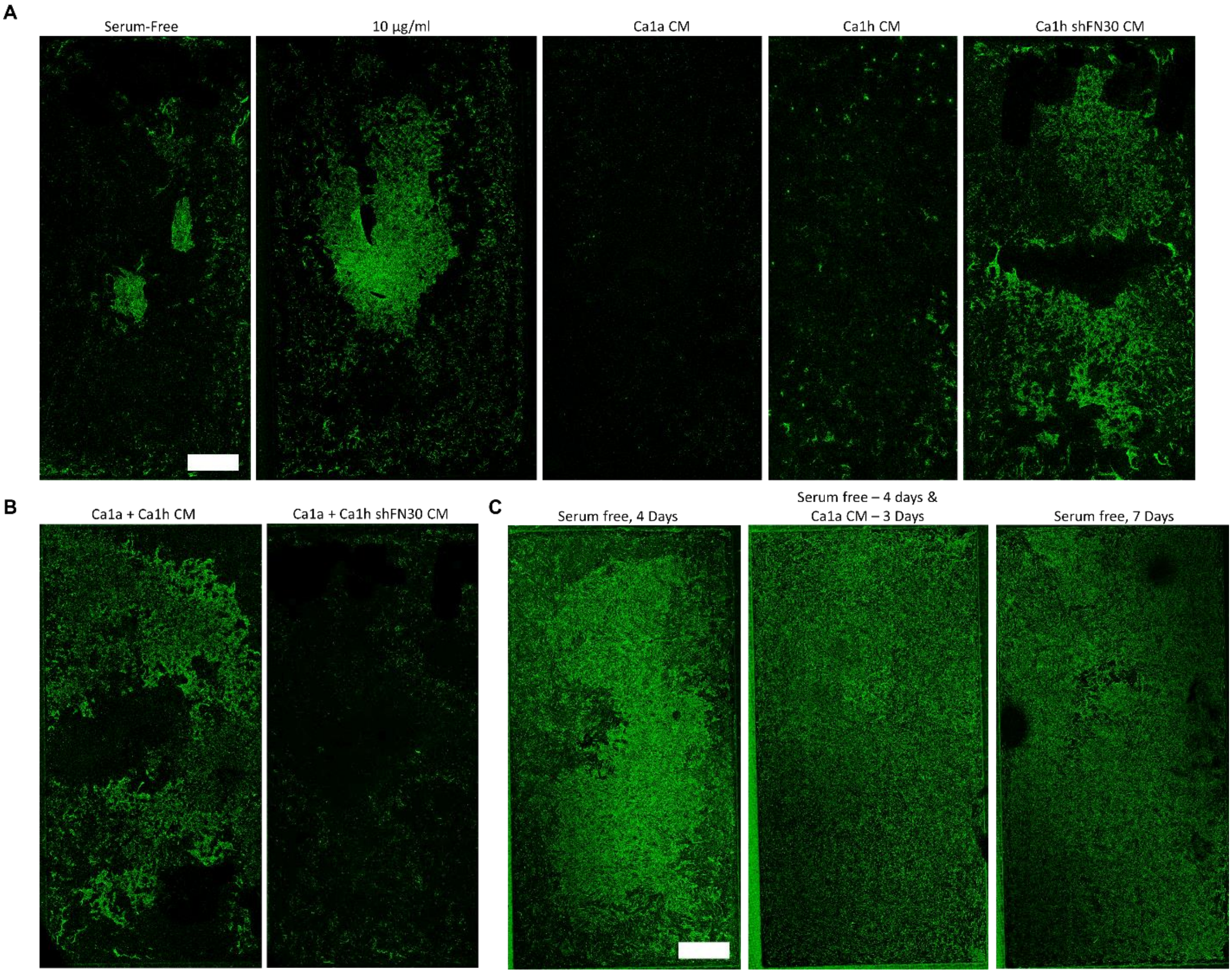

Figure S3. Representative tilescan of FN when HLFs were conditioned with (**A**) endogenous FN, BC CM, or (**B**) CM from BC co-cultures. (**C**) Representative tilescan of FN when partially cultured with Ca1a CM. Scale bar is 1 mm.

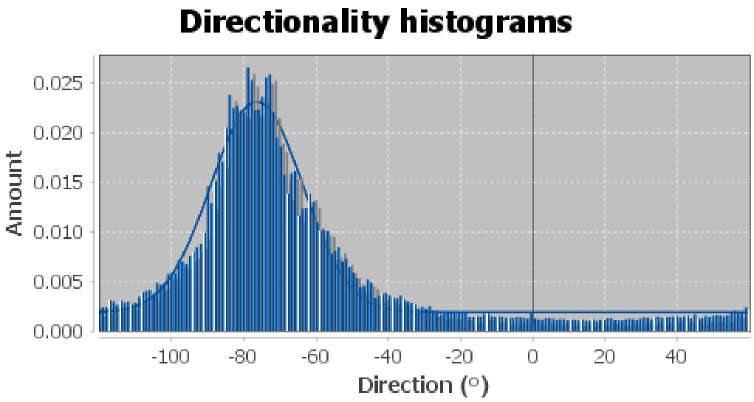

Figure S4. Example directionality histogram for one image in the serum-free HLF group. The amount of fibrils is displayed per direction (180°), binned in 2° increments. The data is fit with a gaussian curve and the dispersion is the standard deviation of the gaussian function.

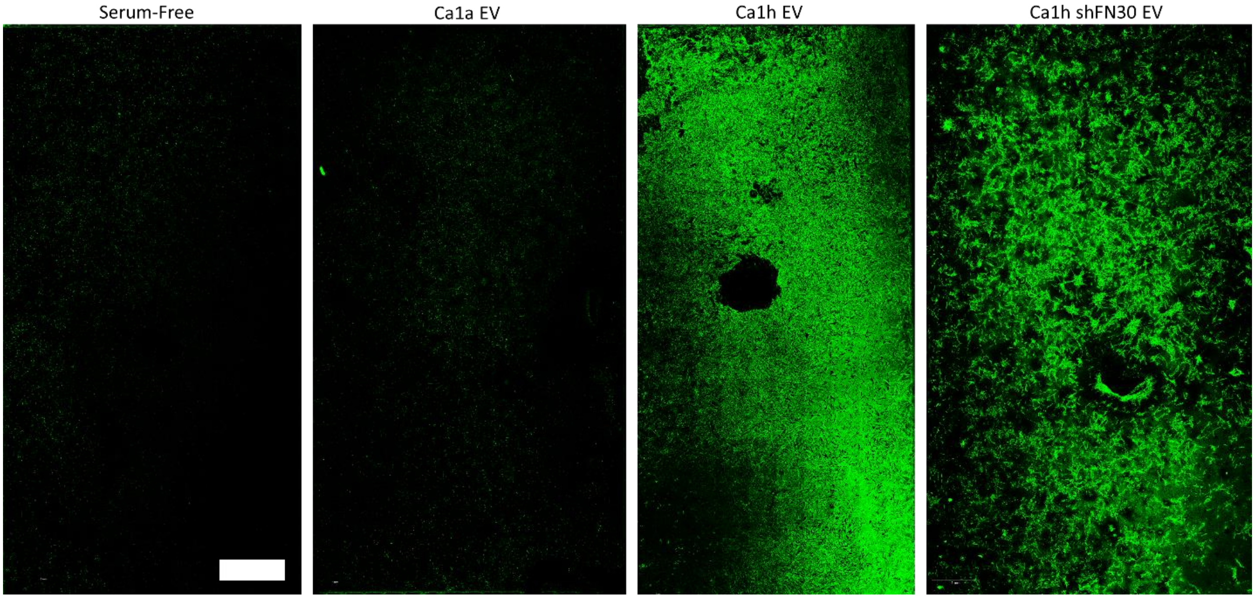

Figure S5. Representative tilescan of FN when HLFs were conditioned with EVs from BC cells. Scale bar is 1 mm.

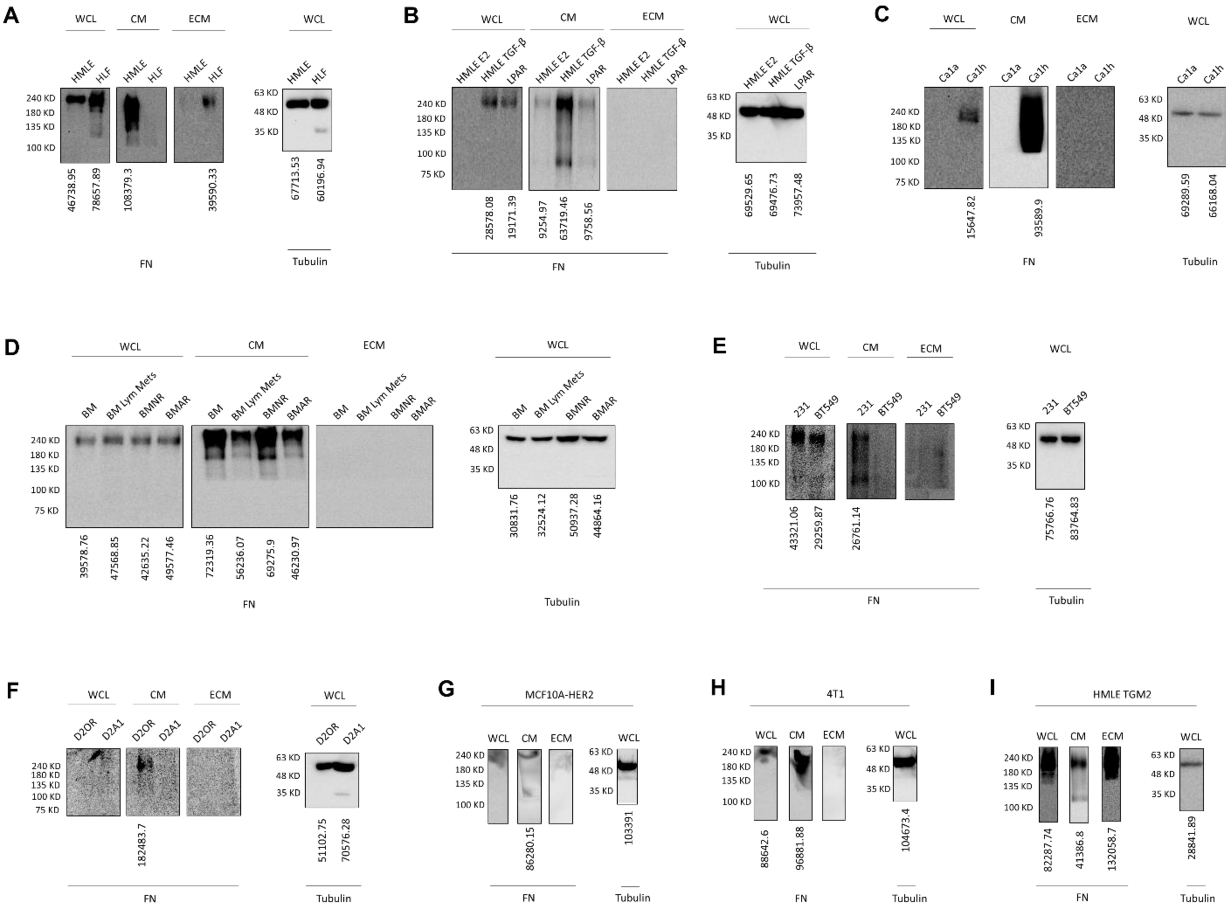

Figure S6. Uncropped immunoblotting of each BC and control cell, grouped by similar lineage, with molecular weight markers and densitometry readings.

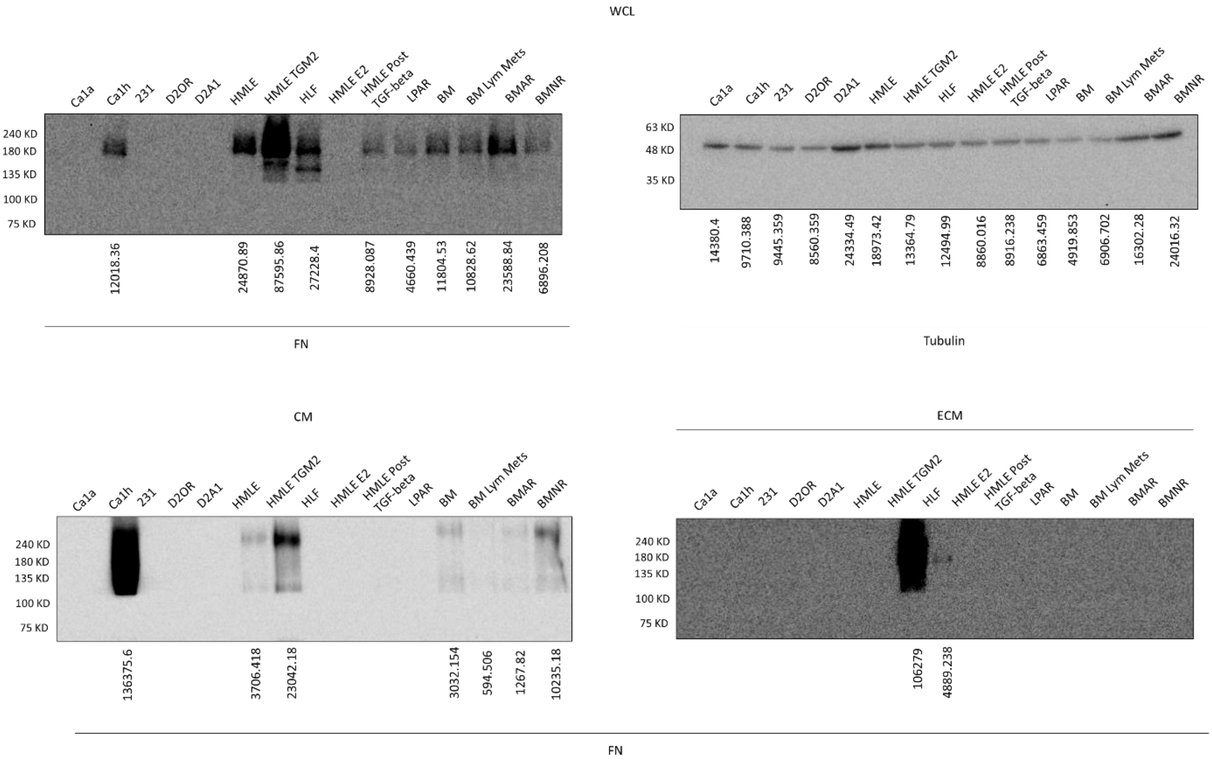

Figure S7. Uncropped combined immunoblotting of the BC and control cells, with molecular weight markers and densitometry readings.

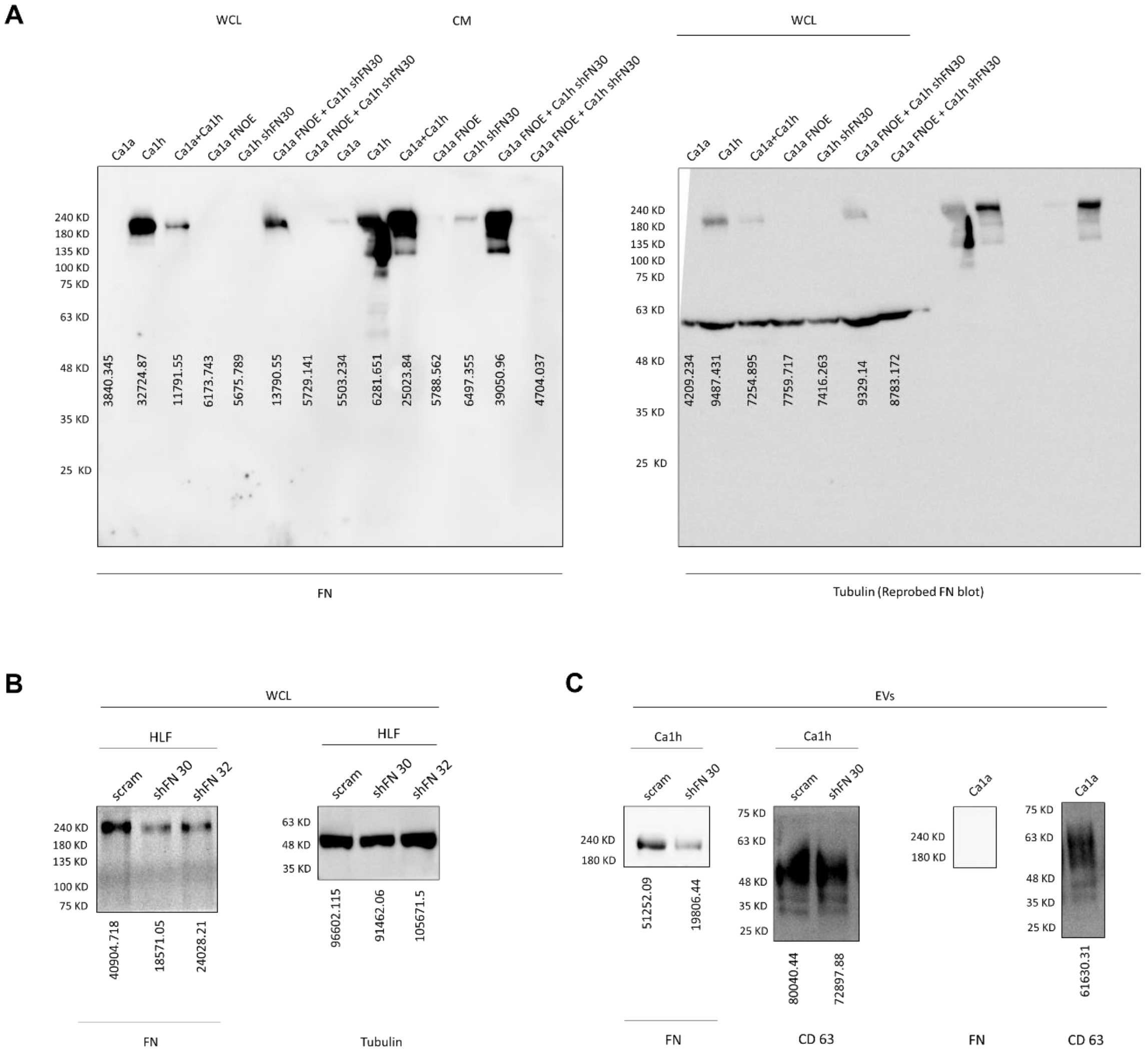

Figure S8. Uncropped immunoblotting with molecular weight markers and densitometry readings for the (**A**) Ca1a, Ca1h, Ca1a FNOE, Ca1h shFN30 cells, and co-cultures, the (**B**) HLFs, and the (**C**) EVs from Ca1h, Ca1h shFN30, and Ca1a cells.

© 2020 by the authors. Licensee MDPI, Basel, Switzerland. This article is an open access article distributed under the terms and conditions of the Creative Commons Attribution (CC BY) license (http://creativecommons.org/licenses/by/4.0/).
